# Construction Notes

**Published:** 1896-12-12

**Authors:** 


### CONSTRUCTION NOTES.

**Children's Ward at the North Devon Infirmary.**

Children hitherto have been accommodated in a ward with adults, but in the future they will have a delightful nursery sanctum to themselves. There are twelve cots in the ward, all of which have been given. Everything likely to brighten the stay of the children has been thought of, and presents of toys, &c., have already been freely made.

**Children's Ward at Barnstaple Infirmary.**

The need of a ward solely devoted to the interests of young children has long been felt at the Barnstaple Infirmary. A partly-used ward at the top of the hospital has been transformed into a lofty, light, cheerful room, pleasantly decorated, and in every way suitable for its purpose. The children's comfort has been considered by the erection of a platform close under the front windows so that they may have a view of the river and the valley in front of the institution. There are twelve cots in the ward, which have been given by friends. With the exception of the structural alterations the whole cost has been met from outside. The new ward is named the "Victoria."

**Extension of Loughborough Hospital and Dispensary.**

Mr. Barrowcliff prepared the plans for the necessary alterations to this institution, an extension in the area of which was urgently needed. The old dispensing-room is being set apart for a nurses' sitting-room, the consulting-room converted into an isolation ward for females, and the waiting-room on the other side of the passage will be an isolation ward for males. The detached laundry and coal buildings in the rear of the hospital have been demolished, and in their place have been erected an out-patients' department and laundry. The structure is imposing, of cream brick, in harmony with the appearance of the hospital proper. The ground floor is devoted to out-patients, leading from the commodious waiting-room, and here is the doctor's consulting-room, which adjoins the dispensing-room. The floors are of wood block. Special attention has been given to the heating, lighting, and general fitting up of the department. What was previously the ironing-room has been ingeniously turned into a ward kitchen. The mortuary remains in its former condition, adjoining it, however, is a new coal-house and jobbing-room. A separate entrance to the cellar has been made, together with a new larder of respectable dimensions. The contractor for the work has been Mr. W. Taitby, of Loughborough, the total cost of which works were estimated at upwards of £600.

